# Anterograde degeneration along the visual pathway following optic nerve injury: a review

**DOI:** 10.3389/fneur.2025.1623798

**Published:** 2025-08-08

**Authors:** Anne-Catherine Chapelle

**Affiliations:** Department of Ophthalmology, Central University Hospital of Liège, University of Liège, Liège, Belgium

**Keywords:** anterograde degeneration, optic neuropathy, multiple sclerosis, Leber hereditary optic neuropathy, amblyopia

## Abstract

The aim of this paper is to review anterograde degeneration throughout the anterior visual pathway, particularly in the optic tracts, the lateral geniculate nucleus, the optic radiations and the visual cortex, following optic nerve injury. Both structural and neuroimaging findings are considered in various diseases, such as optic neuritis and glaucoma. Magnetic resonance imaging findings help localize the specific brain regions where these changes can be observed. Furthermore, particular attention is given to the relationship between cortical atrophy and the location of the associated visual field defect.

## 1 Introduction

The retinal ganglion cells (RGCs) are the optic nerve cell bodies in the retina whose axons converge to form the optic nerve, transmitting visual information to the brain. In neuro-ophthalmology, the analysis of their structural and functional integrity is essential to diagnose and monitor optic neuropathies. Optical coherence tomography (OCT) has emerged as an essential tool in this domain, offering high-resolution scans of retinal layers. Measurement of the ganglion cell complex (GCC) thickness, which includes the ganglion cell layer (GCL) and inner plexiform layer (IPL), provides a sensitive marker of early neuronal damage.

GCC measurements are not only reliable but also remain relatively stable after approximately 3 months after the acute phase of optic neuropathies, such as an episode of non-arteritic anterior ischemic optic neuropathy (NA-AION). Importantly, GCC loss correlates more strongly with the degree of visual field loss than with the degree of peripapillary retinal fiber layer (pRNFL) thinning. Distinct patterns of GCC atrophy may also aid in localising lesion along the visual pathway. For example, GCC thinning in NA-AION often respects the horizontal meridian (1), whereas chiasmal compression typically respects the vertical meridian (2).

Anatomically, RGCs, the third-order neurons of the neuroretina, are much more concentrated in the central retina, with six to seven cell layers, and become sparser in the periphery (3). These cells, which lie between the IPL and the retinal nerve fiber layer, exhibit considerable variable morphologies. RGCs can have either small or large cell bodies (4, 5). Larger ganglion cells are typically found in the periphery, while smaller ganglion cells are located in the perifoveal region of the macula (6). These cells serve as projection neurons of visual stimuli. Their axons follow the inner surface of the retina, converge at the optic disc, and form the optic nerve.

The majority of RGC axons terminate in the lateral geniculate nucleus (LGN) and are classified based on the LGN layers in which they terminate. For instance, parvocellular-type (P-type) ganglion cells terminate in the parvocellular layers (layers 3–6) and magnocellular-type (M-type) terminate in the magnocellular layers (layers 1–2) (7). Approximately 10% of the RGC axons project to the subthalamic area. These axons-called melanopsin-containing retinal ganglion cells-play a role in the pupillary light reflex and circadian rhythm regulation.

While optic neuropathies are often viewed as localized to the anterior visual pathway, growing evidence suggests that RCG damage can lead to widespread neurodegenerative change along the visual pathway. It has been shown that a close relationship exists between the optic nerve and the brain. Indeed, recent evidence suggests that optic neuropathies cause neuronal degeneration in the LGN and visual cortex. Two types of axon degeneration are described: anterograde and retrograde degeneration. *Anterograde degeneration* (8) is a phenomenon that occurs when the axon distal to the site of injury degenerates (i.e., from the cell body of the nerve toward the visual cortex), whereas *retrograde degeneration* refers to damage that leads to the loss of the proximal axon and cell body (i.e., from the cortex back to the cell body of the nerve). These bilateral phenomenon rase the concept of “bidirectional transsynaptic axonal degeneration” that has redefined how we understand the consecutive change after optic nerve injury (9). Historically, Waller was the first to describe distal axonal degeneration in the 19th century, based on his observations of followingglossopharyngeal and hypoglossal nerves transection in frogs (10). This was later termed “Wallerian degeneration,” now widely recognizes as a classical model of anterograde change within the affected neuron (11).

Experimental studies have been key in elucidating the cellular and molecular mechanisms behind this process. For example, the work by Georges and Griffin using a dorsal radiculotomy rat model demonstrated structural degeneration in both the central and the peripheral nervous system (12). Their findings revealed several changes such as granular disintegration of the cytoskeleton, clearance of axonal and myelin debris, blood-tissue-barrier disruption and macrophage recruitment. Macrophages were shown to facilitate myelin clearance and promote neural regeneration - processes with therapeutic relevance to diseases such as multiple sclerosis (MS) (11, 13) and Parkinson’s disease (14).

This review aims to synthesize current knowledge on anterograde transsynaptic degeneration in optic neuropathies, examining its anatomical basis, clinical implications and experimental evidence.

## 2 Anatomical considerations and transsynaptic axonal degeneration model in the visual system

The visual system is particularly well suited for studying neurodegenerative processes as its pathways are anatomically well defined. In the central nervous system, three major pathways arise from the retina: the retinogeniculocalcarine, retinomesencephalic, and retinohypothalamic pathways (15). Each serves distinct roles in visual and non-visual functions, and all are vulnerable to transsynaptic degeneration (TSD) following optic nerve injury.

**Figure 1:**
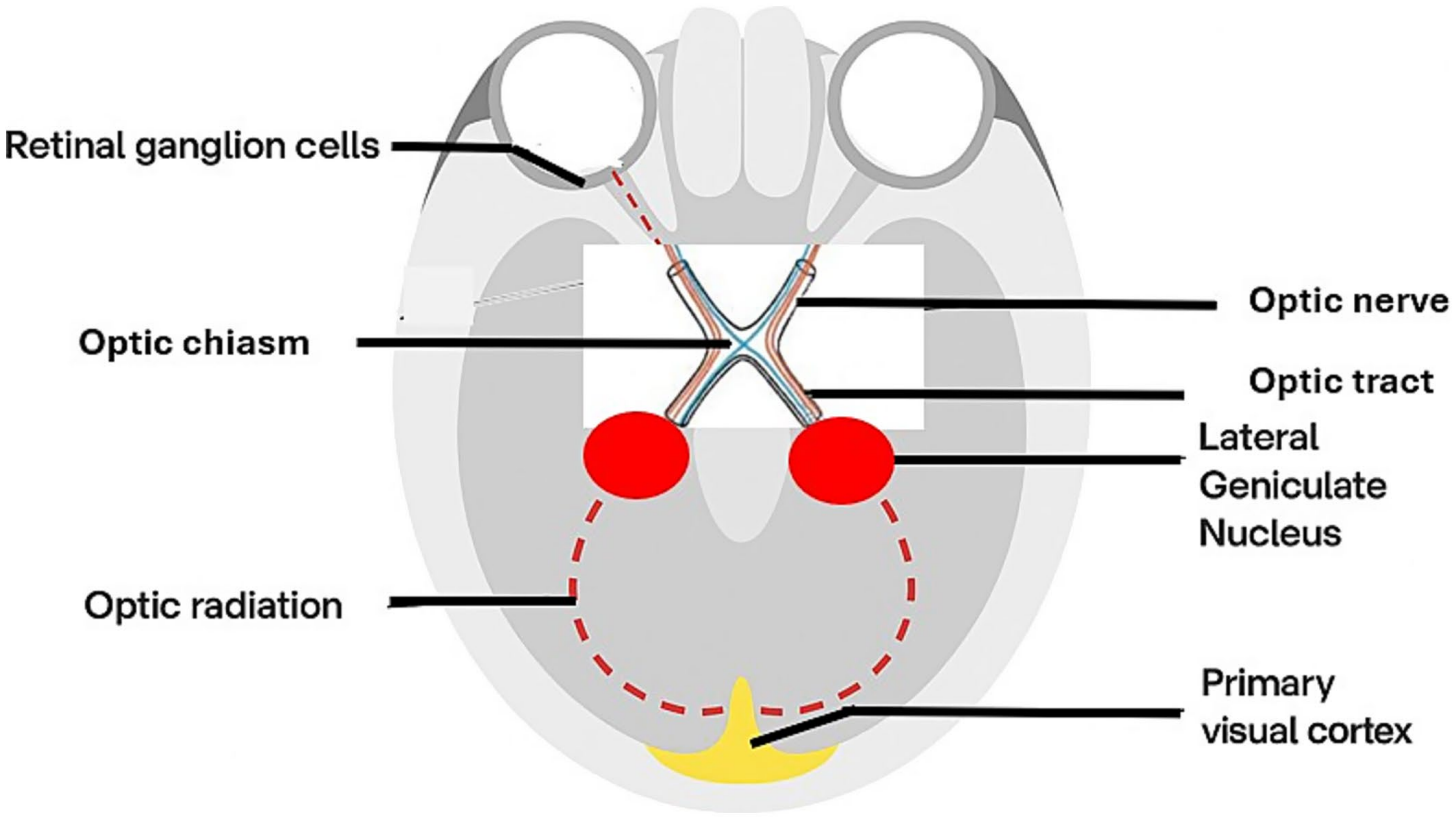
Illustration of the *retinogeniculocalcarine pathway.*

The *retinogeniculocalcarine pathway* is the primary pathway for image-forming visual processing. Visual information from the retina travels along the optic nerve, partially decussates at the optic chiasm, before forming the optic tract and projecting to the LGN of the thalamus. From the LGN, second-order neurons project via the optic radiations to the primary visual cortex (V1), completing the classical visual pathway (Figure 1).

The pupillary light reflex is supported by the *retinomesencephalic tract*, which carries axons from the retina to the midbrain pretectal area, ultimately activating both Edinger–Westphal nuclei and oculomotor nerves (cranial nerve III).

Finally, the retinohypothalamic projection mediates circadian rhythm and contributes to mood regulation by innervating neurons within the suprachiasmatic nucleus of the hypothalamus via melanopsin-containing retinal ganglion cells (16). Notably, these RGCs also contribute to the pupillary light response, linking visual input with physiological rhythms.

Given these interconnected pathways, injury to the optic nerve can result in TSD in these downstream structures-including the LGN, the visual cortex, the pretectal midbrain, and the suprachiasmatic nucleus. It highlights the broader implications of localized RCG injury, extending well beyond the retina. This anatomical model provides a functional framework for exploring the mechanisms and clinical implications of TSD across the visual system.

Despite these clear anatomical relationships, studying TSD *in vivo* remains technically challenging. Many of the affected structures lie deep within the brain and are small in volume, making them difficult to assess using conventional imaging. These challenges have encouraged interest in advanced neuroimaging techniques that can identify microstructural changes.

## 3 Modern magnetic resonance imaging techniques in the evaluation of visual pathway degeneration

Advances in magnetic resonance imaging (MRI) have revolutionized the study of anterograde degeneration along the visual pathway in patients with optic nerve injury. In TSD, high-resolution imaging modalities are necessary to detect subtle alterations in both gray and white matter structures. Recent technological advances provide better spatial resolution, allowing more precise visualization of degeneration not only at the optic nerve level but also in the LGN, optic radiations and visual cortex.

One of the most widely used method for analyzing gray matter is *voxel-based morphometry* (VBM). This technique enables voxel-wise comparison of local gray matter volume or concentration across subjects (17). VBM is particularly useful for detecting volumetric loss in structures such as the LGN and cortical visual areas.

To study white matter integrity, which is particularly important for analyzing the optic tracts and radiations, *diffusion tensor imaging* (DTI) is the most commonly employed technique (18). DTI provides an indirect estimation of fiber integrity by measuring anisotropic water diffusion (assess the directionality of water diffusion). A key metric derived from DTI - fractional anisotropy (FA) - serves as an indirect marker of axonal integrity and myelination.

Building on DTI data, *fiber tractography* enables three-dimensional reconstruction of white matter tracts based on the data collected via DTI (19). For instance, it offers visualization and quantitative analysis of specific tracts such as the optic radiations. This has proven especially useful in tracking the progression of TSD over time in both clinical and research settings.

However, the DTI technique has limitations in differentiating crossing from non-crossing fibers within a single voxel, which can lead to reduced accuracy in region of complex fiber architecture. To address this issue, a more advanced method called *fixel-based analysis* (FBA) has been developed (20). A “fixel” refers to a specific fiber population within a voxel, distinguished by its orientation (21). FBA can therefore differentiate subpopulations of fibers based on their orientation. Several metrics are used in this analysis: fiber density (FD, reflecting intra-axonal volume), fiber-bundle cross-section (FC, indicating macrostructural changes), and a combined metric offering a comprehensive view of micro-and macrostructural integrity-fiber density and cross-section (FDC) (21).

These advanced imaging techniques have transformed the way we understand and monitor the neurodegenerative cascade that can follow optic nerve injury. They are summarized in Table 1, which provides a comparative overview of these imaging modalities, summarizing their principles, anatomic targets and clinical applications.

**Table 1:** Comparative overview of magnetic resonance imaging modalities which summarize their principles, anatomical targets and clinical applications.

	Diffusion tensor imaging (DTI)	Voxel-based morphometry (VBM)	Fiber tractography	Fixel–based analysis (FBA)
Method	Mapping the anisotropic movement of water molecules	Comparing anisotropy values	3D-reconstruction technique to assess neural tracts using data collected by DTI	Detect fiber specific white matter alteration
				Fixel: fiber population within a voxel
Part of the brain studied	White matter	White and gray matter	White matter	White matter
Clinical uses	Evaluating white matter abnormalities	Evaluating white and gray matter atrophy on MRI scans	Assessing white matter damages	Assessing microstructural changes like fiber density (FD) and macrostructural changes like fiber-bundle cross section (FC)

## 4 Structural changes in the lateral geniculate nucleus following optic neuropathy

The LGN serves as the primary relay center between the retina and the visual cortex, playing a central role in the propagation of visual information through the central nervous system. Because of its position within the retinogeniculocalcarine pathway the LGN is particularly vulnerable to TSD following optic nerve injury of varying etiologies (22–26) or disruption of downstream connections. Indeed, these structural changes have also been documented in patients with MS lesion in the optic radiations through retrograde transsynaptic degeneration (27). This process highlights the role of the LGN as a critical junction that connects the anterior and posterior visual pathways, making it a common site of degeneration when either upstream (retinal) or downstream (cortical) structures are damaged (24).

Anterograde TSD in the LGN is not limited to one disease entity. For example, in MS-related optic neuritis (MS-ON), high-resolution 3 T MRI has revealed a reduction in LGN volume compared to matched controls (24). Additionally, an association has been observed between GCC thickness and LGN volume, confirming degeneration of post-synaptic targets such as the LGN (24).

Similar findings were reported in patients with neuromyelitis optica spectrum disorder (NMOSD), an inflammatory demyelinating disease associated with optic neuritis (NMO-ON) (22, 23, 25). In a cohort of 39 patients with at least one episode of NMO-ON, LGN volume was quantified using the multiple automatically generated templates (MaGeT) brain segmentation algorithm on 3 T MRI (23). The analysis revealed a mean LGN volume reduction of 3.09 mm^3^ following an episode of NMO-ON (23). As in MS-ON, LGN volume loss in NMO-ON was correlated with both GCC and pRNFL thinning and the number of previous NMO-ON episodes (23). Interestingly, in contrast to MS, no further progressive LGN atrophy was observed in the absence of new inflammatory events (22).

Structural LGN changes have also been reported in Leber hereditary optic neuropathy (LHON), an hereditary mitochondrial disorder causing bilateral optic nerve atrophy (26). Using ultra-high-field 7 T MRI, researchers demonstrated LGN volume reduction in LHON patients, which correlated with pRNFL thickness (26). Post-mortem analysis in two patients of the cohort confirmed the MRI findings (26).

Finally, similar patterns of atrophy have been found in glaucoma, supporting the notion that LGN atrophy is a consequence of anterograde TSD in optic neuropathies. In both normal-tension glaucoma (28) and primary-open angle glaucoma (POAG) (29), LGN volume loss has been correlated with disease severity - as measured by pRNFL thickness.

These findings suggest that MRI could serve as a valuable imaging biomarker across a range of optic neuropathies, regardless of etiology. Taken together, these studies illustrated that the LGN is a dynamic structure susceptible to degenerative change following lesions within the visual pathway.

## 5 Anterograde degeneration along the optic radiations

Following the degeneration of RGCs and subsequent volume loss in the LGN, the optic radiations - which carry visual signals from the LGN to the primary visual cortex - can also undergo TSD. Increasing evidence suggests that optic nerve damage can initiate a degenerative process that affects the optic radiations, even in the absence of direct injury to these fibers.

In glaucoma, imaging studies have demonstrated atrophy of the optic tracts, optic radiations, and occipital cortex, particularly in patients with advanced disease (21, 30, 31). Using techniques such as DTI and VBM, researchers have observed decreased gray and white matter volumes in these regions (32, 33). Haykal et al. (21) further confirmed the presence of optic tract and optic radiation atrophy in patients with glaucoma, indicating axonal loss due to TSD. Interestingly, optic tract atrophy was correlated more strongly with advanced glaucoma, whereas optic radiation changes appeared earlier, suggesting a dynamic temporal pattern of degeneration along the visual pathway (21). These results were supported by tractography-based studies showing a three-to four fold reduction in the number of optic radiation fibers projecting to the affected hemifield in patients with POAG (34). These findings highlight how early injury to RGCs can propagate through the visual system.

Similar findings have been made in MS following episodes of optic neuritis. Longitudinal imaging studies have demonstrated progressive damage to the optic radiations, even in the absence of active inflammation (35–38). This anterograde TSD was often more pronounced in patients with severe optic nerve atrophy, suggesting a correlation between anterior and posterior pathway damages. Microstructural analysis using DTI has shown a reduction in FA in the optic radiations, indicating ongoing axonal degeneration (35). Finally, Manogaran et al. demonstrated a positive correlation between optic radiation myelin water fraction in the optic radiations and both pRNFL and GCL thicknesses in MS-ON and NMO-ON (39).

Similar degeneration has been observed in LHON. Imaging studies have reported volume loss in the optic radiations (40), providing additional evidence of anterograde TSD following optic nerve injury.

These findings highlight the vulnerability of the optic radiations to anterograde TSD after damage to the optic nerve. They also reinforce the importance of imaging these tracts in patients with optic neuropathies-to monitor disease progression and assess the effectiveness of potential neuroprotective therapies.

## 6 Degeneration along the lateral geniculate nucleus projections to the extrastriate visual cortex

While V1 is the principal target of the optic radiations, visual information is distributed from V1 to multiple extrastriate areas responsible for higher-order functions such as motion perception, object recognition and visuospatial integration. As such, degeneration originating from the optic nerve and propagating through the LGN and the optic radiations, can affect not only the primary visual cortex (V1) but also the other associationvisual areas.

One of the clearest examples of anterograde TSD is found in POAG. Post-mortem studies have shown atrophy in V1 that reflects the topography of the visual field defect. For example, a case report revealed thinning of the cortex below the calcarine sulcus in a patient with a superior visual field defect due to POAG (41). This finding has been confirmed by other MRI studies (32, 42, 43). In POAG, volume reductions have been identified not only in primary but also in secondary visual cortices and association areas, including the inferior fronto-occipital fasciculus, parietal lobules, and dorsal parietal and occipital areas, all of which are involved in visuospatial processing (30, 42, 44). In addition to atrophy around the calcarine sulcus, the left middle temporal gyrus and fusiform gyrus have also been shown to exhibit volume loss (32). These regions are all implicated in visuospatial attention and motion processing.

Similarly, in optic neuritis, both primary and secondary cortical changes have been observed. Audoin et al. reported reduced gray matter magnetization transfer ratio - a marker of myelin integrity—in the visual cortex of patients with prior MS-ON (45). Balk et al. (9) further confirmed primary visual cortex atrophy following MS-ON and demonstrated a strong correlation between optic nerve atrophy (pRNFL and GCC thinning) and cortical atrophy in V1, reinforcing the idea that degeneration travels anterogradely along the visual pathway.

In NMOSD, Tian et al. (25) demonstrated volume loss in V1 following an episode of NMO-ON, consistent with patterns observed in MS. Notably, this cortical atrophy was found even in the absence of new lesions or inflammatory activity, supporting the hypothesis that chronic degenerative processes continue after the initial optic nerve involvement.

Even in retinal diseases, such as albinism and hereditary retinal dystrophies, MRI studies have also identified structural alterations in the occipital cortex. For example, authors have demonstrated reduced surface area of the calcarine fissure (46) and reduced gray matter volumes in the occipital pole (47) of individuals with albinism. Similarly, Plank et al. reported occipital atrophy in hereditary retinal dystrophies (48). These findings emphasize the relationship between the location of visual field defects and the topography of cortical degeneration but also the principle that chronic retinal degeneration leads to structural reorganization of the brain.

Despite these advances, some extrastriate areas remain understudied. One notable example is extrastriate area V5, which is critical for motion processing (49). Although it is a major projection target of the LGN, little is known about how optic nerve damage affects this region. Given the functional relevance of V5, further investigation is needed, especially in conditions where patients report motion-related visual disturbances.

## 7 Experimental evidence of anterograde degeneration in animal models

While clinical imaging has provided compelling evidence of anterograde TSD in humans following an optic neuropathy, animal models remain essential for elucidating the longitudinal course and the molecular mechanisms underlying this phenomenon. Animal studies have confirmed that damage to the optic nerve initiates a degenerative cascade that extends to the LGN and V1, mirroring the anatomical progression seen in humans.

### 7.1 Primate models

Assessing anterograde TSD in primates is particularly challenging - not only due to its extended time course but also because of key anatomical differences between the primate and human visual systems.

Vickers et al. induced glaucomatous optic neuropathy by elevating intraocular pressure (IOP) in one eye of experimental animals (50). Histological analysis revealed a reduction in cytochrome oxidase staining and decreased synaptophysin immunolabelling in both the parvocellular and magnocellular layers of the LGN receiving input from the affected eye (50). These findings were supported by another study that demonstrated a 35% reduction in the magnocellular layer and a 23% reduction in the parvocellular layer of the LGN following 2.5 weeks of increased IOP (51).

Further analysis confirmed that damage extended beyond the LGN to involve V1, particularly a subdivision of the layer IV known as layer IVC, which receives direct input from the LGN. This cortical degeneration in the layer IVC was particularly evident in the ocular dominance columns corresponding to the damaged eye, as indicated by reduced cytochrome oxidase staining (50). Notably, the extent of cortical involvement was strongly correlated with the severity of RGC loss rather than with elevated IOP alone (52), highlighting that neuronal input, rather than pressure change, drives anterograde TSD.

### 7.2 Rodent models

Although the rodent visual system differs markedly from that of primates and humans – particularly in the proportion of retinal projections to subcortical structures, it offers powerful tools for molecular dissection. In rodents, approximately 95% of RGC axons project contralaterally to retinorecipient nuclei, including the superior colliculus, the suprachiasmatic nucleus, the dorsal LGN (dLGN), the ventral LGN, and the intergeniculate leaflet (33, 53).

In one study, researchers performed optic nerve transection 2–3 mm posterior to the globe in rats (33) and apoptotic changes were observed in the dLGN as early as 1 week after injury, with progression noted at one-and two-months post-lesion. Interestingly, apoptosis in V1 followed a similar timeline (33), suggesting that neuronal loss in post-geniculate structures is a delayed but integral part of the transsynaptic degenerative process.

At the molecular level Akt dephosphorylation was identified as the earliest molecular sign of degeneration in both the dLGN and V1 (33). This change occurred as early as 1 week after optic nerve transection and appeared to initiate the molecular cascade leading to apoptosis in anterograde TSD (33). These findings offer potential molecular targets for neuroprotective interventions aimed at halting or reversing transsynaptic damage.

### 7.3 Comparison of experimental models and human observations

Overall, these experimental studies mirror the clinical observations made in optic neuropathies such as glaucoma, MS-ON and LHON. They provide strong biological validation for the concept of anterograde TSD.

However, the temporal dynamics and extent of degeneration vary between species. For example, cortical changes in primates evolve over weeks to months, similar to human disease, while rodent models tend to exhibit more rapid progression. Following optic nerve injury in rodents, axonal degeneration of the LGN is typically observed within 1 week, followed by degeneration of the optic radiations between 1 to 5 months, and subsequently involving the visual cortex - most commonly between 3 to 5 months post-injury (54). Intriguingly, this study showed two subpopulations that may be more or less susceptible to injury outcomes (54).

## 8 Anterograde degeneration differences after damage occurring prenatally or in childhood versus in adulthood

The developing visual system is highly plastic and early-life ocular pathology can significantly disrupt the normal development of visual pathways. The timing of the optic nerve injury-whether in childhood or adulthood - plays a critical role in determining the pattern andextent of anterograde degeneration and the amount of compensation that occurs.

### 8.1 Anterograde degeneration in amblyopia

During childhood, the visual cortex undergoes rapid synaptogenesis and its maturation is heavily dependent on normal retinal input. Visual experience during this critical period shapes cortical architecture and any disruption – such as amblyopia caused by strabismus or refractive error - can lead to cortical alterations.

MRI studies in children with amblyopia exhibited decreased gray matter volumes in V1 and extrastriate visual cortex compared to healthy controls (55, 56). These volumetric reductions in visual areas were more pronounced in pediatric cases compared to adults (57), suggesting that early visual deprivation leads to more profound and potentially irreversible cortical changes. In adults with amblyopia, decreased gray matter volume has also been noted in the LGN (58). In cases of unilateral amblyopia, a difference between the two hemispheres cortical thickness was shown (59). These volume losses were primarily located in areas responsible for spatial vision (60).

### 8.2 Insights from enucleation studies

The impact of early versus late visual loss has also been examined in individuals who underwent unilateral enucleation - typically due to retinoblastoma. In a longitudinal MRI study, Kelly et al. observed reduced volume in the LGN and optic tract ipsilateral to the enucleated eye, along with milder but measurable atrophy contralaterally (61). These changes reflect the redistribution of inputs and outputs across the visual pathway.

Interestingly, a similar pattern of degeneration was observed in an adult patient who underwent enucleation at age 50 due to trauma (61). However, in contrast to pediatric patients, the degree of LGN atrophy was significantly more severe, suggesting that neuroplasticity may afford greater protection when injury occurs early in life.

These developmental differences have serious implications for both diagnosis and treatment. In children, early identification and correction of visual pathway insults (for example, amblyopia or congenital cataract) may prevent or minimize anterograde TSD (62, 63).

## 9 Discussion

The visual system offers a unique and valuable model for studying anterograde degeneration in the central nervous system. This pathway allows researchers to trace the consequences of localized injury-such as optic neuropathy - across multiple neural levels with precision.

Across a broad range of optic neuropathies - including MS-ON, NMO-ON, LHON, and glaucoma-, evidence shows that RGC loss initiates a cascade of degenerative changes that extend to the LGN, optic radiations and ultimately the visual cortex.

The integration of emerging MRI techniques, particularly DTI, advanced tractography, FBA and VBM has enabled clinicians and researchers to noninvasively map these changes *in vivo*. These methods offer sensitive and specific biomarkers for disease monitoring and prognosis. Animal models have validated these observations and provided insights into the cellular and molecular events underlying TSD. They have clarified that degeneration is an active regulated process involving early metabolic change and apoptotic pathways.

Despite significant progress, important questions remain. The impact of optic nerve injury on subcortical structures - such as pretectal area involved in the pupillary reflex and the suprachiasmatic nucleus involved in circadian regulation - have not been explored. Additionally, extrastriate areas such as V5, which is central to motion perception, remain under investigated in the context of optic neuropathies. Future studies are needed to evaluate if V5 exhibits change similar to that observed in V1.

## Glossary

dLGN: Dorsal Lateral Geniculate Nucleus
DTI: Diffusion Tensor Imaging
FBA: Fixel-Based Analysis
FC: Fiber-Bundle Cross-Section
FD: Fiber Density
FDC: Fiber Density and Cross-section
GCC: Ganglion cell complex (GanglionCell Layer (GCL) + Inner Plexiform Layer (IPL))
IOP: IntraOcular Pressure
LGN: Lateral Geniculate Nucleus
LHON: Leber Hereditary Optic Neuropathy
MRI: Magnetic Resonance Imaging
MS-ON: Multiple Sclerosis-related Optic Neuritis
NA-AION: Non-Arteritic Anterior Ischemic Optic Neuropathy
NMO-ON: Neuromyelitis Optica Spectrum Disorder associated with optic neuritis
NMOSD: Neuromyelitis Optica Spectrum Disorder
OCT: Optical Coherence Tomography
POAG: Primary Open-Angle Glaucoma
pRNFL: peripapillary Retinal Nerve Fiber Layer
RGC: Retinal Ganglion cell
V1: Primary Visual Cortex
VBM: Voxel-Based Morphometry
